# The Role of Vitamin D3 Deficiency and Colonization of the Oral Mucosa by *Candida* Yeast-like Fungi in the Pathomechanism of Psoriasis

**DOI:** 10.3390/jcm13226874

**Published:** 2024-11-15

**Authors:** Mariola Marchlewicz, Paulina Sagan, Marta Grabowska, Magdalena Kiedrowicz, Joanna Kruk, Kamil Gill, Małgorzata Piasecka, Ewa Duchnik

**Affiliations:** 1 Department of Dermatology and Venereology, Faculty of Health Sciences, Pomeranian Medical University in Szczecin, 70-010 Police, Poland; mariola.marchlewicz@pum.edu.pl (M.M.); wietrakp@wp.pl (P.S.); magdalena.kiedrowicz@pum.edu.pl (M.K.); 2 Department of Histology and Developmental Biology, Faculty of Health Sciences, Pomeranian Medical University, 71-210 Szczecin, Poland; kamil.gill@pum.edu.pl (K.G.); malgorzata.piasecka@pum.edu.pl (M.P.); 3 Faculty of Physical Culture and Health, University of Szczecin, 71-065 Szczecin, Poland; joanna-szczecin@wp.pl; 4 Department of Aesthetic Dermatology, Faculty of Health Sciences, Pomeranian Medical University in Szczecin, 70-111 Szczecin, Poland; ewa.duchnik@pum.edu.pl

**Keywords:** psoriasis, vitamin D3, oral mucosa, *Candida*

## Abstract

Psoriasis is a chronic inflammatory skin disease with complex pathogenesis and variable severity. Performed studies have indicated the impact of vitamin D3 deficiency on the pathogenesis of psoriasis and its severity. However, there is no clear evidence of the influence of the mucosal microbiome on the onset and progression of psoriasis. This review aims to present the current evidence on the role of vitamin D3 and colonization of the oral mucosa by *Candida* yeast-like fungi in the pathogenesis of psoriasis. *Candida albicans* is a common yeast that can colonize the skin and mucosal surfaces, particularly in individuals with weakened immune systems or compromised skin barriers. In psoriasis, the skin’s barrier function is disrupted, potentially making patients more susceptible to fungal infections such as *Candida*. Since patients with psoriasis are at increased risk of metabolic syndrome, they may experience the vicious circle effect in which chronic inflammation leads to obesity. Vitamin D3 deficiency is also associated with microbiological imbalance, which may promote excessive growth of *Candida* fungi. Under normal conditions, the intestinal and oral microflora support the immune system. Vitamin D3 deficiency, however, leads to disruption of this balance, which allows *Candida* to overgrow and develop infections.

## 1. Introduction

Psoriasis is a chronic inflammatory skin condition characterized by periods of remission and flare-ups, with incidence estimated at approximately 2% of the general population. It has a broad spectrum of clinical manifestations and severity. Psoriasis can develop at any age and its course differs depending on many extrinsic and intrinsic factors [1].

The incidence of psoriasis varies in different geographical regions. For example, in the US and Western Europe, approximately 2% of the population is affected [2]. Two peaks of the age of onset have been identified: the first during puberty and the second at the age of 50–70 years [3].

Psoriasis has a complex pathogenesis and varying severity [1,2,3]. Chronic inflammation in psoriasis may lead to systemic complications, in particular metabolic disorders. They are associated with an increased cardiovascular risk, deficiency of vitamins and microelements, musculoskeletal conditions, and, consequently, deteriorated overall quality of life and increased mortality [1]. Studies have indicated the impact of vitamin D3 deficiency on the pathogenesis of psoriasis and its severity [4]. However, there is no clear evidence of the influence of the skin and mucosal microbiome on the onset and progression of psoriasis [5].

This connection seems to be important for several reasons. *Candida albicans* is a common yeast that can colonize the skin and mucosal surfaces, particularly in individuals with weakened immune systems or compromised skin barriers, such as in psoriasis patients. In psoriasis, the skin’s barrier function is disrupted, potentially making patients more susceptible to fungal infections such as *Candida* [6,7,8].

Investigating this link could provide insights into whether *Candida* acts as a trigger or exacerbating factor for psoriasis flares. If a correlation is found, treating *Candida* infections might offer a novel therapeutic pathway to reduce the severity of psoriasis. Additionally, understanding the interaction between fungal colonization and immune responses in psoriasis could shed light on the broader mechanisms driving both skin inflammation and the disease’s progression. Finally, it may help to identify subgroups of psoriasis patients who could benefit from antifungal treatments alongside traditional therapies, potentially improving outcomes and quality of life.

Additionally, the literature in this field provides evidence of the association between diet, physical activity (PA), oxidative stress (OS), and psoriasis [9,10,11].

Although several studies have been conducted, there is still limited information concerning the role of vitamin D3 in psoriasis development, treatment, and prevalence. Thus, this review aims to present the current evidence on the role of vitamin D3 and colonization of the oral mucosa by *Candida* yeast-like fungi in the pathogenesis of psoriasis. Moreover, this review seeks to prove the relationship between vitamin D3 deficiency and the risk of colonization by yeast-like fungi *Candida*.

## 2. Characteristics of Psoriasis

### 2.1. Pathogenesis

The development of psoriatic lesions is strongly associated with chronic inflammation leading to the abnormal differentiation and proliferation of keratinocytes. A characteristic feature of this disease is an abnormally fast migration of keratinocytes from the dermis to the epidermis [12]. T cells play a dominant role in the inflammatory infiltrate of psoriatic skin. CD4+ cells are more abundant in the dermis, while CD8+ cells are mainly found in the epidermis [13].

It is believed that the onset of psoriasis is associated with a trigger that induces chronic inflammation, in which the interaction of keratinocytes with immune cells plays a fundamental role. Previous studies have suggested that psoriatic lesions primarily involve the epidermis, but now it is clear that the chronic nature of the condition is also attributed to interactions between immune cells in the dermis. Trauma is a widely recognized factor that may trigger the clinical onset of psoriasis. Patients develop characteristic psoriatic lesions in injured areas of the skin. This phenomenon is called the Koebner reaction and occurs especially in cases of epidermal trauma. It has been speculated that the enhanced capillary blood flow in the dermal papillae stimulated by trauma facilitates the migration of inflammatory mediators into the epidermis [14]. A marked upregulation of the nerve growth factor (NGF) in Koebner-positive lesions and epidermotropism of T cells was reported [15]. Skin trauma also induces an increased secretion of interferon α (IFNα) and IFNβ in keratinocytes [16].

**Figure 1:**
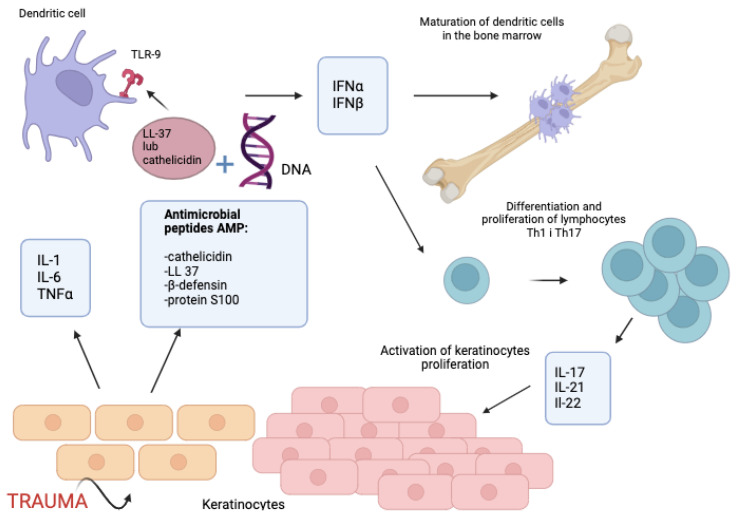
Pathogenesis of psoriasis; part I. Created with BioRender.com. When keratinocytes are exposed to trauma, they release various inflammatory mediators such as interleukin-1 (IL-1), IL-6, tumor necrosis factor α (TNFα), chemokines, and antimicrobial peptides (CAMPs), including cathelicidin (LL-37), β-defensin, and S100 proteins. In pathological conditions such as psoriasis, LL-37 or cathelicidin can bind to host DNA. This binding activates the toll-like receptor 9 (TLR-9) on dendritic cells, which then stimulates an immune response. Interferons IFNα and IFNβ secreted by keratinocytes play a role in the maturation of dendritic cells in the bone marrow and are responsible for the differentiation and proliferation of Th1 and Th17 cells, which secrete cytokines such as IL-17, IL-21, and IL-22.

The inflammatory pathway leading to the development of psoriatic lesions is complicated and not fully explained. In response to trauma, keratinocytes secrete numerous inflammatory substances such as interleukin-1 (IL-1), IL-6, tumour necrosis factor α (TNFα), chemokines, and cationic antimicrobial peptides (CAMPs), including cathelicidin (LL-37), β-defensin, and S100 protein [17,18,19]. Under physiological conditions, CAMPs bind to anionic lipid components of the bacterial walls and cause damage. Under pathological conditions, LL-37 or cathelicidin binds to the deoxyribonucleic acid (DNA) of the host and stimulates the toll-like receptor 9 (TLR-9) on the surface of dendritic cells, leading to their stimulation and increased secretion of IFNα and IFNβ. These two interferons stimulate the maturation of dendritic cells residing in the bone marrow and are responsible for the differentiation and proliferation of Th1 and Th17 cells. Th cells are mainly responsible for enhanced proliferation of keratinocytes in the epidermal psoriatic lesions by secreting cytokines, in particular IL-17, IL-21, and IL-22 [19] (Figure 1).

**Figure 2:**
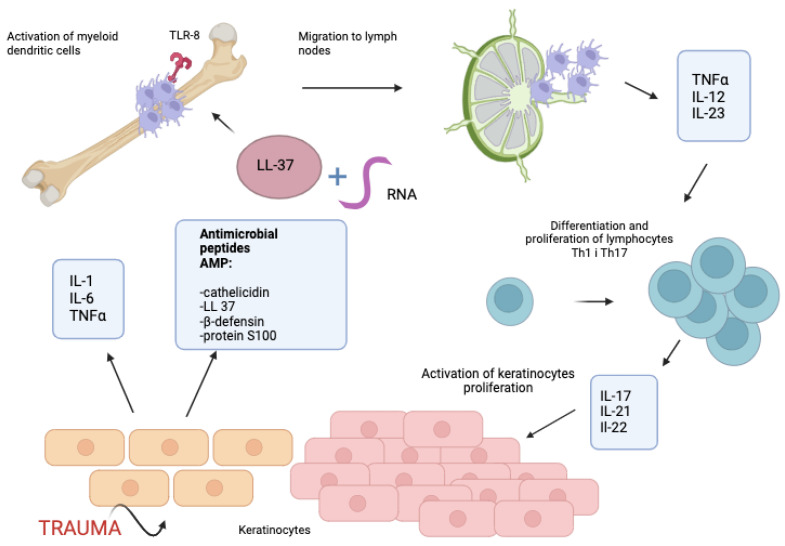
Pathogenesis of psoriasis; part II. Created with BioRender.com. When keratinocytes are exposed to trauma, they release various inflammatory mediators such as interleukin-1 (IL-1), IL-6, tumor necrosis factor α (TNFα), chemokines, and antimicrobial peptides (CAMPs), including cathelicidin (LL-37), β-defensin, and S100 proteins. In pathological conditions such as psoriasis, LL-37 can bind to host RNA. This binding activates the toll-like receptor 8 (TLR-8) on dendritic cells, which then stimulates migration of dendritic cells to lymph nodes and the secretion of TNFα, IL-12, and IL-23. This results in the differentiation and proliferation of Th1 and Th17 cells, which secrete cytokines such as IL-17, IL-21, and IL-22.

Another complex involved in the pathogenesis of psoriasis is formed by LL-37 (released from stimulated keratinocytes) and ribonucleic acid (RNA). This complex binds to the TLR-8 receptor and activates myeloid dendritic cells. In response, dendritic cells migrate to the lymph nodes and secrete TNFα, IL-12, and IL-23. Similarly to the above-described activation of TLR-9, this pathway is also responsible for the activation of Th1 and Th17 cells. Moreover, the cathelicidin–RNA complex stimulates slan+ monocytes residing in the psoriatic skin, which secrete pro-inflammatory cytokines [19,20] (Figure 2).

Genetic factors play a significant role in the pathogenesis of psoriasis. The polymorphism of the major histocompatibility complex (MHC) has been reported as particularly important in the development of this disease. The HLA-C*06:02 allele (human leukocyte antigen, HLA-Cw6) was found in approximately 60% of psoriasis patients [21]. Psoriasis was also reported to be associated with numerous single-nucleotide polymorphisms, e.g., IL-23 and IL-23 receptor [22]. There is also a proven association between psoriasis and mutations in the gene encoding caspase recruitment domain-containing protein 14 (CARD 14). Mutations in IL36RN, encoding the IL-36 receptor antagonist, is a risk factor for generalized pustular psoriasis [23]. A relationship between epigenetic factors, such as micro-ribonucleic acid (miRNA) and gene methylation and the risk of psoriasis has also been demonstrated [24].

An interesting area of research on the pathogenesis of psoriasis concerns neutrophil extracellular traps (NETs), first described in 2004. These structures released by neutrophils are formed from decondensed chromatin fibres, histone proteins, and antibacterial enzymes. NETs may be formed in a lytic or non-lytic process. Under physiological conditions, NETs are released in response to pathogens, in particular to bacterial lipopolysaccharides or polysaccharides in the mycelium wall [25]. In patients with psoriasis, neutrophils are preactivated. Through released proteins as well as reactive oxygen species (ROS) and associated oxidative stress (OS), neutrophil extracellular traps may lead to the disturbance of redox homeostasis [9]. Stimulated keratinocytes produce cationic antimicrobial proteins, including cathelicidin, which further forms the above-described pathogenic complexes by interacting with chromatin released from neutrophils (catehelicidin–autoDNA complex) [26]. Another component of neutrophil extracellular traps is neutrophil gelatinase-associated lipocalin, called lipocalin-2. Levels of lipocalin-2 are higher in psoriasis patients and correlated with the severity of itching [27,28]. Hu et al. [26] reported that an increased NET formation in peripheral blood and psoriatic skin biopsies correlated with the severity of psoriasis assessed by the PASI score.

### 2.2. Risk Factors

The extrinsic risk factors for psoriasis include bacterial, viral, and fungal infections. The relationship between the onset of psoriasis and streptococcal infection has been well documented. Guttate psoriasis frequently follows bacterial infections [29]. A study by Pietrzak et al. also demonstrated that detection rates of *Candida* spp., mainly *C. albicans*, in the oral mucosa of patients with psoriasis were significantly higher compared to healthy subjects [30]. Other pathogens that may trigger psoriatic lesions include *Staphylococcus aureus*, *Malassezia* spp., human immunodeficiency virus (HIV), human papilloma virus (HPV), and retroviruses [31,32,33,34].

Another important risk factor for psoriasis is lifestyle (unhealthy diet, low level of PA, smoking, psychological stress, and alcohol consumption) and related lifestyle diseases such as dyslipidemia, hypertension, obesity, and type 2 diabetes [35,36]. It is well documented that an anti-inflammatory diet, such as a diet rich in vegetables and fruits, healthy fats, lean protein, and whole grains, making use of spices and herbs, as well as limiting alcohol intake and not smoking fight inflammation, decrease the risk factor for developing psoriasis, and help ease some psoriasis outcomes [10,11,37,38,39]. In addition, a healthy and balanced diet combined with regular PA can cause weight loss and may contribute to a reduced risk of obesity.

It has also been speculated that exposure to air pollutants may have a significant negative effect on the skin. Compounds such as polycyclic aromatic hydrocarbons, volatile organic compounds, oxides, ozone, heavy metals, and ultraviolet radiation cause skin damage and enhance ROS production [32,40].

Psoriasis can also be triggered or exacerbated by certain types of medications, including beta-blockers, lithium, antimalarials, interferon, imiquimod, angiotensin-converting enzyme inhibitors, terbinafine, tetracycline, nonsteroidal anti-inflammatory drugs, and fibrates [32,41,42,43].

During the COVID-19 pandemic caused by the SARS-CoV-2 infection, psoriasis exacerbation was reported in some patients who recovered from SARS-CoV-2 infection and after COVID-19 vaccination [44].

### 2.3. Clinical Manifestations and Treatment

Plaque psoriasis is the most commonly diagnosed type, representing approximately 90% of psoriatic patients. It is a chronic form with periods of flare-ups and remissions. The lesions are usually characterized by sharply demarcated erythematous plaques covered with scales [45]. Skin involvement varies, from small patches to the presence of multiple plaques covering large areas of the body [46]. Lesions are usually located on the abdomen, on the extensor surfaces of the extremities, and the scalp [47].

Other less common clinical forms of psoriasis include inverse, guttate, pustular, or erythrodermic [46,48,49,50]. Psoriatic arthritis is diagnosed in approximately 20% of patients [51]. Nail psoriasis may accompany skin and joint lesions or be an isolated symptom of the disease. It develops in about 50–80% patients with psoriasis [52].

It is impossible to cure psoriasis, but a complete remission of the disease can be achieved with modern therapies in many patients. Topical treatment is recommended for mild plaque psoriasis. This therapy is chosen because of the low risk of side effects. The most popular topical medications include keratolytic agents, dithranol, glucocorticoids, and vitamin D analogues [46]. Systemic treatment is used primarily in patients with moderate-to-severe psoriasis (with active PA), generalized pustular psoriasis, and psoriatic erythrodermia [53]. The most popular systemic therapies for psoriasis include photochemotherapy, methotrexate, retinoids, cyclosporine A, biologics, phosphodiesterase 4 inhibitor, and inhibitors of Janus-activated kinases.

## 3. Vitamin D3

### 3.1. Physiological Effects of Vitamin D3

**Figure 3:**
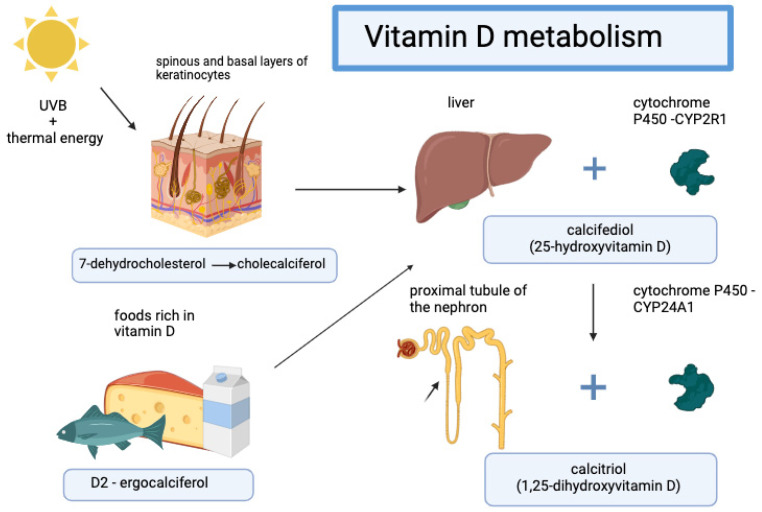
Vitamin D metabolism. Created with BioRender.com. Vitamin D3 is produced from 7-dehydrocholesterol in the epidermis under UVB exposure, converting into cholecalciferol. Both vitamin D2 and D3 bind to DBP and are transported to the liver, where they convert to 25-hydroxyvitamin D (25(OH)D3 or calcidiol) via CYP2R1. This inactive form is then transported to the kidney’s proximal tubule, where it is hydroxylated by CYP24A1 at position 1 to form calcitriol [1,25(OH)2D3] or by CYP27B1 at position 24 to form 24,25(OH)2D3.

The two major forms of vitamin D are vitamin D2 (ergocalciferol) and vitamin D3 (cholecalciferol). In plants and mushrooms, vitamin D2 is produced from ergosterol after irradiation with UVB light. Vitamin D3 is produced from 7-dehydrocholesterol present in the epidermis, located in the cell membranes of the spinous and basal layers of keratinocytes, which after exposure to UVB is converted into lumisterol and then tachysterol. These compounds undergo thermal isomerization, and cholecalciferol is formed [54]. Vitamin D2 and D3 combined with vitamin D-binding protein (DBP) are transported with blood to the liver and in reaction with cytochrome P450 (CYP2R1) are converted to 25-hydroxyvitamin D [25 (OH) D3], or calcifediol. The inactive form, 25 (OH) D3, is transported to the epithelium of the proximal renal tubule, and in reaction with CYP24A1 is hydroxylated at position 1 to form calcitriol: [1,25 (OH)2 D3] or hydroxylated in reaction with CYP27B1 at position 24 to form 24,25 (OH)2 D3 (Figure 3).

This process depends on the concentration of many substances, including calcium, phosphorus, parathormone, fibroblast growth factor (FGF), and Klotho protein [54,55].

Calcitriol is an active form of vitamin D, playing an important role in the skeletal, immune, and cardiovascular systems. Like other steroid hormones, calcitriol acts on effector cells. It binds to the vitamin D receptor (VDR), which first forms a heterodimer with the retinoid X receptor and then binds to the relevant DNA sequences. Hundreds of genes and DNA sequences involved in calcium–phosphate metabolism, as well as having extraskeletal significance have been identified in effector cells [56]. Calcitriol is also involved in epigenetic mechanisms by binding to cellular membrane proteins. This process results in the activation of intracellular proteases and kinases and the production of prostaglandins by the cells. These compounds are responsible for the effects of vitamin D on the epigenome [57].

### 3.2. The Effects of Vitamin D3 on the Metabolism and on Cutaneous and Mucosal Immune Systems

The skin plays an important role in the production of vitamin D3, and its supplementation supports the treatment of psoriasis and other skin conditions due to the modulation of the inflammatory response. Vitamin D activates or suppresses gene transcription via the intracellular pathway that involves the nuclear vitamin D receptor—9-cis retinoic acid receptor VDR-RXR [58]. The active form of vitamin D plays a role in the differentiation and proliferation of keratinocytes in the basal layer of the skin. It also enhances the synthesis of keratin 1 and 10, and is essential in the regulation of apoptosis. Vitamin D inhibits the proliferation of B and T cells, including CD25+/CD4+ Tregs, and induces the expression of the C-C chemokine receptor type 10 on the surface of T cells [59].

Considering the pathogenesis of psoriasis, vitamin D3 stimulates the expression of the previously discussed CAMPs (e.g., cathelicidin), which have antiviral and antifungal properties. It should be noted that the immune response to microbial pathogens must be balanced to prevent generalized inflammatory tissue damage. It has been suggested that the cathelicidin–vitamin D3 axis is important in an adequate inflammatory response. Therefore, vitamin D3 may activate the secretion of CAMPs but also suppress the immune response [59,60]. Vitamin D also enhances the synthesis of involucrin, transglutaminase, loricrin, and filaggrin in the spinous layer of the epidermis, and regulates the synthesis of glycosylceramide, which is responsible for the integrity of the epidermal barrier and the permeability of the epidermis. Calcitriol stimulates the synthesis of ceramide by inducing sphingomyelinase (thereby increasing the conversion of sphingomyelin to ceramide), and in return ceramide enhances the pro-differentiating effect of calcitriol on keratinocytes. Calcitriol also improves the integrity of the stratum corneum and normalizes the distribution of integrins [59].

Vitamin D3 deficiency leads to dysregulated secretion of pro-inflammatory cytokines, such as TNFα, IL-1ß IL-6, and IL-8. Many researchers have emphasized the significant antioxidant function of vitamin D3. It is speculated that vitamin D3 plays an important role in the prevention of autoimmune diseases by inhibiting the differentiation of B cells and their transformation into plasma cells [59]. This may reduce the level of circulating serum antibodies.

Because of the broad range of its regulatory effects on the immune system, vitamin D3 has been used in topical treatment of psoriasis, especially in combination with glucocorticosteroids. A systematic review of studies demonstrated the synergistic treatment effect of calcipotriol combined with corticosteroid, as well as a reduced side effect profile [61].

A significant relationship between the serum concentration of vitamin D3 and gastrointestinal diseases was reported recently. For example, an adequate level of vitamin D is an important factor in improving the composition of the gut microbiota. It prevents epithelial barrier dysfunction that leads to increasing gut permeability and damage by bacterial antigens, and improves the integrity of intestinal epithelial cells. The antibacterial effect of vitamin D3 consists mainly in stimulating the expression of proteins responsible for antigen recognition on the cell surface and enhanced secretion of anti-inflammatory peptides by intestinal cells [62].

In summary, vitamin D3 plays a vital role in skin health, particularly concerning conditions such as psoriasis and other inflammatory disorders. It promotes the differentiation of keratinocytes, enhancing keratin production, which is crucial for maintaining the skin barrier. Vitamin D3 also stimulates the synthesis of antimicrobial peptides, bolstering the skin’s defence against pathogens [19,59]. Moreover, it influences immune responses by modulating the activity of various immune cells, including T cells and dendritic cells. Vitamin D3 can enhance the production of cytokines that regulate inflammation, potentially reducing the inflammatory response in skin conditions. It also promotes the expression of CAMPs (cathelicidin antimicrobial peptides), which play a key role in skin immunity [17,18,19]. Additionally, vitamin D3 activates the vitamin D receptor (VDR), which interacts with genes involved in skin cell proliferation and immune function, further supporting skin health and homeostasis [56,58,59].

### 3.3. Vitamin D3 Levels in Patients with Psoriasis

Some studies have reported vitamin D3 deficiency in patients with psoriasis vulgaris [59,63]. So far, no clear explanation for this relationship has been provided, but the immunomodulating and normalizing effect of vitamin D3 on keratinocytes discussed in the previous section may indicate the role of vitamin D3 deficiency in the pathogenesis of psoriasis. Vitamin D3 may also prevent an inadequate immune response and the development of autoimmune diseases by suppressing the expression of TLRs [64]. As discussed above, the activation of TLR-9 on plasmacytoid dendritic cells is fundamental in a complex inflammatory pathway that results in the stimulation of Th1 and Th17 cells and the secretion of different interleukins [19]. This may explain the postulated impact of vitamin D3 deficiency on the development of other autoimmune diseases, including type 1 diabetes, multiple sclerosis, inflammatory bowel diseases, rheumatoid arthritis, and autoimmune thyroiditis [65]. The hypothesis on the role of vitamin D3 deficiency in the pathogenesis of psoriasis may be supported by the fact that the A-1012G polymorphism of the VDR receptor gene associated with lower VDR mRNA expression is more frequently detected in psoriasis patients [59].

Another study demonstrated that a BMI > 27 is an additional risk factor for vitamin D3 deficiency in psoriasis patients [63]. Vitamin D3 deficiency was also reported in patients with simple obesity [66]. Despite the proven role of vitamin D in the physiology of the psoriatic skin, the evidence of beneficial effects of oral vitamin D supplementation remains controversial [59].

Research suggests that vitamin D, once stored in adipocytes, is less likely to be mobilized into the bloodstream, where it can exert its physiological effects. The high volume of adipose tissue acts as a reservoir, limiting the release of vitamin D into circulation and thus reducing the overall bioavailability of both the parent compound (cholecalciferol) and its active metabolite (calcitriol), which is essential for calcium homeostasis and immune regulation [66]. Additionally, adipose tissue is known to secrete inflammatory cytokines that can further exacerbate this issue by increasing systemic inflammation. Chronic inflammation is known to alter the metabolism of vitamin D, potentially impairing its conversion to the active form (1,25-dihydroxyvitamin D) in the kidneys and liver. This is particularly relevant in the context of psoriasis, where inflammation is already elevated, compounding the problem of vitamin D deficiency and making the skin condition more difficult to manage [67,68].

Moreover, studies have shown that vitamin D stored in fat is less responsive to oral supplementation, meaning that even higher doses of vitamin D may not be sufficient to restore normal blood levels in obese individuals. This is due to the increased volume of adipose tissue acting as a sink for the vitamin, reducing its bioavailability for other tissues, including the skin, bones, and the immune system, all of which are critical targets of vitamin D’s biological effects [66,69].

### 3.4. Psoriasis and Physical Activity

A relatively small number of studies have focused on the effect of PA on skin diseases, including psoriasis, compared to research on the correlation between diet and psoriasis outcomes [11].

The existing literature has well documented that regular moderate-to-vigorous physical activity (defined as a metabolic equivalent task (MET) of 3 to ≤9 METs) exhibits a wide range of benefits on physical and mental health, such as promoting weight loss, reducing inflammation, correcting metabolism, and improving well-being [11,70,71]. Regulation of ROS levels, prevention of OS via enhanced expression of genes participating in antioxidant production, and effects on cells’ adaptation to OS are essential properties of PA related to protection against skin diseases, including the development and severity of psoriasis [11,70,71,72,73,74].

Another important property of PA includes its effects on skin moisturizing and physical barrier function [75]. Both effects are essential for human health. Emerging evidence suggests that the regulation of the release of water from the body to the atmosphere and the skin’s function as a barrier preventing the invasion of pathogens are caused by dysfunction of mitochondrial DNA [76]. Evidence shows that PA can stimulate mitochondrial synthesis, increasing stratum corneum hydration [75]. The findings showed that individuals with moderate and high PA habits experienced increased skin moisturizing function compared to individuals with low PA habits. However, there is no clear consensus in the literature on which type of exercise and intensity are the most effective for skin moisturizing and barrier function. Ryosuke et al. suggested that long-term endurance PA may improve the skin structure, whereas a single endurance exercise may reduce stratum corneum hydration [75].

Other essential protective mechanisms of PA include elevated serum levels of vitamin D3, regardless of whether individuals exercise outdoors or indoors [77]. Research has indicated that PA habit levels are significantly positively correlated with circulating metabolites of vitamin D3: 1.25(OH)2D3 and 25(OH)D3 [78]. It has been shown that endurance training in PA, but not resistance training, increased the serum 25(OH)D level in individuals with vitamin D3 deficiency, whereas only chronic endurance PA increased serum 1.25(OH)2D3 metabolite. The research suggests that the effect of PA on serum vitamin D3 levels is multifactorial, including the vitamin content in the diet [78]. This suggestion may explain why results on this topic are inconclusive. A quantification of the association of psoriasis with PA showed that patients with severe psoriasis practice vigorous exercise 32% less often than individuals without psoriasis, and undertook regular PA 12% less often [10]. Moreover, patients with psoriasis had reduced sports activities [73], and over 50% of them did not practice PA at the dose recommended by the WHO for good health [79]. Meanwhile, the current literature presents a reduced risk of psoriasis development by approximately 30% among individuals practising regular vigorous PA (≥6–9 METs). Referring to the benefits of PA in psoriasis, Yeh et al. [80] reported that engaging in vigorous aerobic exercise and calisthenics more than 4 h per week reduced the thinning of the stratum corneum, increased Pgc-1α expression (the agent responsible for mitochondrial biogenesis), and lowered the risk of psoriasis.

The authors reported that running 105 min weekly was associated with a 25–30% reduced risk of psoriasis compared with sedentary behaviour.

### 3.5. The Pathomechanism of Metabolic Syndrome as a Potential Cause of Vitamin D3 Deficiency in Psoriasis Patients

Patients with psoriasis have an increased risk of metabolic syndrome (MS). MS is not a separate disease but a cluster of clinical conditions leading to increased mortality from cardiovascular causes. According to the 2009 consensus statement, these conditions include arterial hypertension, abdominal obesity, hyperglyceridaemia, low levels of high-density lipoprotein (HDL), and carbohydrate metabolism disorders. Metabolic syndrome is diagnosed in patients who have at least three out of these five conditions. MS is associated with about a three-fold higher risk of cardiovascular diseases and a five-fold higher risk of type 2 diabetes [81].

A relationship between the PASI score and the risk of metabolic syndrome was reported [82]. There is also evidence for a relationship between the body mass index (BMI) and a risk of psoriasis [83]. A positive correlation was demonstrated between waist circumference and the occurrence and severity of psoriasis [84]. The above data indicate that psoriasis patients require comprehensive care and assessment of cardiovascular risk.

The pathomechanism of metabolic syndrome in psoriasis is complicated and involves genetic and environmental factors. Undoubtedly, insulin resistance is fundamental to the development of metabolic syndrome. Currently, chronic inflammation mediated by cytokines (IL-17, TNFα, adiponectin, IL-6) is implied in the development of insulin resistance [85]. It is known that psoriasis patients suffer from chronic inflammation and have higher levels of proinflammatory cytokines compared to healthy people. Moreover, higher leptin levels have been reported in the blood and tissues of patients with psoriasis. Leptin plays an important role in regulating metabolism and is a satiety factor [86]. Leptin resistance is found in obese patients due to its persistently elevated levels. The influence of oxidative stress accompanying skin inflammation and intestinal microflora disorders on the development of MS in patients with psoriasis has also been suggested [87].

There are several possible reasons for vitamin D3 deficiency in obese patients. The first one is a lower exposure to sunlight. A more probable explanation, however, is the fact that vitamin D3, as a fat-soluble compound, is accumulated in adipocytes. Vitamin D3 circulating in the plasma is accumulated in adipose tissue and becomes less bioavailable. This was confirmed by lower concentrations of vitamin D3 in the blood in obese patients compared to people with normal BMI after oral supplementation with the same dose of vitamin D3 [66].

Since patients with psoriasis are at an increased risk of metabolic syndrome, it can be assumed that they may experience the vicious circle effect in which chronic inflammation leads to obesity. Obesity further leads to vitamin D3 deficiency, which at a later stage contributes to the exacerbation of the primary disease. Weight loss seems to resolve skin lesions in patients with psoriasis and improves their sensitivity to systemic therapies, allowing for reduction of drug doses and thus reducing their side effects [85].

In summary, the pathomechanism of metabolic syndrome in psoriasis is multifaceted, involving genetic and environmental factors. Chronic inflammation driven by cytokines (such as IL-17, TNFα, and IL-6) exacerbates insulin resistance, which plays a key role. Psoriasis patients have higher levels of these pro-inflammatory cytokines compared to healthy individuals. Moreover, leptin, a hormone involved in metabolism regulation, is found in higher concentrations in psoriasis patients, suggesting its role in the disease’s metabolic and inflammatory processes [67,85].

A beneficial effect of vitamin D3 on the skin in patients with psoriasis has been reported [88]. However, due to the limited number of studies and significant differences in the doses of vitamin D3 administered to patients, no optimal daily supplementation dose has been proposed to date [59]. It is worth noting that sufficient serum levels of vitamin D can prevent a psoriasis flare up when a trigger occur [89].

## 4. *Candida* Yeast-like Fungi

### 4.1. Candida spp. as a Commensal and Pathogen

Yeast-like *Candida* fungi were isolated for the first time in 1844 from the sputum of a patient with tuberculosis [90]. Among the numerous species, *Candida albicans* is the most common commensal found in the mucous membranes of the human gastrointestinal tract. Epidemiological data indicated that up to 80% of the population had their oral cavity colonized by *C. albicans* [91]. However, in immunocompromised patients, it may cause opportunistic mucosal infections and even candidemia, with a mortality risk of up to 70%. In developed countries, *Candida* spp. is the third or fourth most common cause of candidemia in hospitalized patients [6]. The transformation of *Candida* spp. into an invasive form is a complex process and includes changes in the morphology from the yeast-like form, which consists of single blastospores, to the hyphal form [7]. The invasive form shows adherence to epithelial cells and thigmotropic behaviour. Hyphal cells have the ability of directional growth in response to contact with a surface (thigmotropism), allowing the fungus to invade intercellular junctions [91]. Moreover, the biofilm formation by *Candida albicans* plays a key role in the growth of the invasive form [92]. This process has four steps. The first step is the adhesion of hyphal cells to the host tissues. In the second step, biofilm development continues and the number of cells increases. In the third step, extracellular polymeric substances are secreted, forming an extracellular matrix which creates a scaffold for biofilm as well as a connection with the host cells [93]. The final fourth step is the dispersal, in which mature biofilms release non-adherent daughter cells to propagate infection. The entire biofilm formation cycle usually takes from 24 to 48 h [92,93]. Biofilm is a special pathogenic factor because it is responsible for resistance to treatment and recurrent infections [92]. At the same time, it constitutes an in vivo structure facilitating bacterial infections [94]. Biofilm can cover the surface of medical equipment such as catheters, prostheses, and pacemakers, posing a particular risk in patients with many comorbidities and compromised immunity [95].

Risk factors for candidiasis include quantitative and qualitative reductions of saliva, dentures, topical and systemic corticosteroid therapy, smoking, broad-spectrum antibiotic therapy, old age, immunodeficiencies, and malnutrition [96,97,98]. Oral candidiasis has two clinical forms, acute and chronic. The acute form includes acute pseudomembranous candidiasis (thrush) and acute atrophic candidiasis, including the erosive variant. Chronic forms of candidiasis include chronic atrophic candidiasis, chronic hyperplastic candidiasis, angular cheilitis, often diagnosed in children and in patients after a stroke, and median rhomboid glossitis [54]. Another chronic but rare condition is cheilo-candidiasis [91,99].

Healthy people have adaptive immunity to *Candida* spp. colonizing the oral mucosa. It is unclear why the full immune response is triggered by the progressing infection. One explanation may come from the fact that the hyphal form of *Candida* contains more immunogenic oligosaccharides in the cell membrane, which are involved in the stimulation of human immune cells. Both morphological forms of *Candida* spp. stimulate the immune system in different ways. The yeast-like form activates TLR-4 receptors and stimulates the secretion of INFγ by activating Th1 cells, while the hyphal form activates the immune system through dectin-1 [100].

Immunological tolerance to the yeast-like form of *Candida* involves a limited response of the immune system. The response is strong enough to control the growth of yeast-like cells but not to cause local inflammation. This homeostasis is continued and involves epithelial cells, lymphocytes, neutrophils, and macrophages. C-type lectin receptors, in particular dectin-2, seem to play a special role in the immune response to the yeast-like *Candida* spp., limiting its invasion [101]. Dectin-2, present on dendritic cells, monocytes, and macrophages, has a high affinity for mannose-like structures and is involved in the activation of Th17 cells. Subsequently, Th17 cells produce IL-17, which recruits neutrophils to protect the host mucosa [102].

Evidence for the role of Th17 cells and IL-17 in immunity to *Candida* spp. has been provided from studies on patients with chronic mucocutaneous candidiasis. These patients have a genetic defect in the Th17 cell-dependent secretion of IL-17 and IL-22 [103]. The role of IL-17 in response to the colonization of the oral mucosa with *Candida* spp. seems particularly interesting in psoriasis exacerbated by this pro-inflammatory cytokine or other factors.

### 4.2. The Colonization of the Oral Mucosa by Candida Yeast-like Fungi in Psoriasis Patients

A meta-analysis by Liang et al. revealed a higher detection rate of *Candida* spp. in patients with psoriasis compared to healthy people, and its positive correlation with the PASI score [8].

There are many hypotheses on the relationship between *Candida* spp. colonization and the immune response in patients with psoriasis. It has been suggested that toxins released by fungi colonizing the intestine may act as superantigens, non-specifically stimulating Th cells to produce IL-23 and IL-17 and other cytokines [8,104]. Exposure of the skin and mucous membranes to *Candida* spp. via the dectin-2 receptor on immune cells activates Th17 helper cells which further stimulate a Langerhans cell-dependent response [105]. Another mechanism that may mediate the development of psoriasis by *Candida* spp. is NET activation. Neutrophil extracellular traps are formed, for example, in response to *Candida* infections and can stimulate Th17 cells to produce interleukins [106]. Neutrophils can kill the yeast form of C. *albicans* through phagocytosis, but the elimination of the hyphal form of *Candida* requires a broader response of the host’s immune system. It has been demonstrated that beta-glucan, which is the main polysaccharide of the C. *albicans* wall, is recognized by neutrophil beta2-integrin, which results in the nonlytic release of NETs [107]. This process may have a significant impact on the severity of psoriasis through colonization with yeast-like *Candida*. The secretion of NETs in response to other fungal infections is also triggered through dectin-2 [108].

Some clinical studies have shown that the colonization of the skin and mucous membranes by *Candida* spp. is detected more frequently in psoriasis patients than in healthy people. This was reported, for example, in a meta-analysis by Pietrzak et al. [30], which included 1038 psoriasis patients and 669 healthy subjects. The same relationship was also found in a study by Elsner et al. [109], who investigated patients treated and untreated for psoriasis. The study showed higher detection rates of *Candida* spp. in psoriasis patients compared to the control group.

## 5. The Relationship Between Vitamin D3 Deficiency and the Risk of Colonization by Yeast-like Fungi *Candida*

Vitamin D3, although most often associated with the regulation of calcium–phosphate metabolism and bone health, also plays a key role in modulating the immune system. More and more research indicates a strong relationship between vitamin D3 deficiency and increased susceptibility to infections, including fungal infections caused by *Candida* yeasts. These fungi are a natural part of the microbiome of the oral cavity, gastrointestinal tract, and urogenital system, but under favorable conditions, such as a weakened immune system, they can transform into pathogens causing candidiasis.

Vitamin D3, known for its immune-regulating properties, has been shown to play a role in both innate and adaptive immunity. Deficiency in this critical nutrient can weaken the body’s natural defences, leaving individuals more vulnerable to infections, including those caused by fungi like *Candida*. Studies such as those conducted by Lim et al. [110] demonstrated a significant decrease in serum vitamin D3 levels in hospitalized patients who developed *Candida albicans* bloodstream infections compared to those without such infections. This finding points to a direct correlation between low vitamin D3 levels and the heightened risk of invasive *Candida* infections.

Furthermore, in experimental models, vitamin D3 supplementation has been shown to reduce the risk of chronic fungal infections. Lim et al. [110], using mouse models, indicated that vitamin D3 can help modulate immune responses in such a way that it lowers the likelihood of long-term *Candida* colonization. These results suggest that maintaining adequate levels of vitamin D3 may serve as a preventive measure against chronic fungal infections, which often prove challenging to treat.

Another study by Xie et al. [111] in pediatric intensive care units found that supplementing children’s diets with yogurt fortified with vitamin D3 significantly reduced the risk of *Candida*-related infections, such as candidemia and candiduria, and even shortened hospital stays. This insight is critical, especially in light of the growing concern over antifungal resistance, making it harder to treat infections using conventional pharmacotherapy. The ability of vitamin D3 to enhance natural immune defenses could thus play a crucial role in managing fungal infections without over-reliance on drugs.

In addition to these findings, Allemailem [112] observed that vitamin D3 supplementation could improve the efficacy of fluconazole, a common antifungal medication, in animal models. This enhanced effect was linked to the activation of macrophages by the active form of vitamin D3 (1.25 (OH)2 D3), which plays a vital role in boosting the immune response against pathogens, including *Candida*. The phagocytic activity of macrophages, which engulf and destroy fungal cells, was notably increased in the presence of higher levels of vitamin D3. Small et al. [113] observed that when macrophages were cultured in a high-vitamin D3 medium, they displayed increased concentrations of complement receptor immunoglobulin (CRIg), further enhancing their ability to eliminate fungal pathogens.

Lastly, vitamin D3 may exert a direct antifungal effect by altering the integrity of the fungal cell membrane [114]. This property suggests that beyond its immune-modulating abilities, vitamin D3 could directly combat fungal infections at a cellular level, making it a powerful tool in preventing and treating *Candida* infections.

In summary, while more research is needed to fully understand the relationship between vitamin D3 deficiency and *Candida* colonization, the existing evidence points to the critical role of vitamin D3 in supporting immune function and reducing the risk of fungal infections. Supplementation with vitamin D3 could be a promising avenue for preventing *Candida* infections, especially in at-risk populations, and may enhance the effectiveness of current antifungal therapies.

## 6. Conclusions

A beneficial effect of vitamin D3 on psoriatic skin has been demonstrated. So far, the optimal daily supplementation dose of vitamin D3 for psoriasis patients has not been established due to the small number of studies and inconsistent findings.

The main risk factor for secondary vitamin D3 deficiency in psoriasis patients is excess body fat, in which cholecalciferol is sequestered. In the context of psoriasis, where vitamin D plays a role in modulating immune responses and reducing skin inflammation, the sequestration of the vitamin in fat stores can lead to exacerbations of the disease. This highlights the importance of not only addressing vitamin D deficiency through higher doses in obese patients but also considering weight loss as a therapeutic strategy to improve the efficacy of vitamin D and other treatments for psoriasis. Obesity may be associated with a more severe course of psoriasis, and the secretion of IL-17 by adipocytes is among its causes. Furthermore, severe psoriasis is a risk factor for the development of metabolic syndrome.

*Candida* infections, such as mucosal candidiasis, can become chronic, especially in people with vitamin D3 deficiency. Chronic infections are more difficult to treat because of *Candida* ability to form biofilms, which protect the fungi from the effects of antifungal drugs and the immune response. Vitamin D3 plays an important role in preventing such conditions by stimulating the immune response, including the activation of macrophages and lymphocytes, which eliminate pathogens more effectively. Vitamin D3 deficiency is also associated with microbiological imbalance, which may promote excessive growth of *Candida* fungi. Under normal conditions, the intestinal and oral microflora support the immune system, preventing the development of pathogens. Vitamin D3 deficiency, however, leads to disruption of this balance, which allows *Candida* to overgrow and develop full-blown infections. Currently, there is no clear evidence for higher detection rates of *Candida* spp. in patients with untreated psoriasis. The higher frequency of colonization by *Candida* spp. observed in some studies may be primarily due to the pharmacotherapy.

It is worth mentioning that current evidence supports the concept that regular vigorous PA, due to its anti-inflammatory properties, obesity prevention, skin moisturizing, and influence on increased release of vitamin D3, can be beneficial in counteracting the development and severity of psoriasis.

## 7. Future Direction

Cholecalciferol supplementation is recommended by the Polish Endocrine Society for all individuals during autumn and winter [115]. However, in the light of available literature data, psoriasis patients seem to benefit particularly from such treatment. Therefore, it would be justified to introduce vitamin D3 supplementation in the treatment of psoriasis and monitor vitamin D3 levels in order to adjust the adequate supplementation dose. However, it seems that for economic reasons this option will still not be widely available in the coming years. In the current situation, an alternative approach may be the supplementation of a standard dose in patients following updated recommendations of international scientific societies, with particular emphasis on their age and body weight, as well as increasing individuals’ awareness of the benefits of regular PA. In this respect, further research should validate the type and dose of exercise training, based on accurate and objective measures of exercise components, and identify more precise biochemical mechanisms operating between PE and psoriasis.

A multicentre and long-term prospective study is required to clearly answer the question of whether more frequent colonization by yeast-like *Candida* spp. fungi influences the development of psoriasis. The study design would have to consider healthy individuals and detection rates of *Candida* spp. on mucous membranes and then long-term follow-up focused on the development of psoriasis. However, such a study is expected to be costly, difficult to organize, and seems unfeasible at the moment.

## Data Availability

All relevant data are presented in the article.
